# Overexpressing STAMP2 Improves Insulin Resistance in Diabetic ApoE^−/−^/LDLR^−/−^ Mice via Macrophage Polarization Shift in Adipose Tissues

**DOI:** 10.1371/journal.pone.0078903

**Published:** 2013-11-13

**Authors:** Lu Han, Meng-Xiong Tang, Yun Ti, Zhi-Hao Wang, Jia Wang, Wen-Yuan Ding, Hua Wang, Yun Zhang, Wei Zhang, Ming Zhong

**Affiliations:** 1 Key Laboratory of Cardiovascular Remodeling and Function Research Chinese Ministry of Education and Chinese Ministry of Public Health, Ji’nan, People’s Republic of China; 2 Department of Cardiology, Qilu Hospital of Shandong University, Ji’nan, People’s Republic of China; 3 Department of Emergency, Qilu Hospital of Shandong University, Ji’nan, People’s Republic of China; 4 Department of Geriatric Medicine, Qilu Hospital of Shandong University, Ji’nan, People’s Republic of China; University of Tor Vergata, Italy

## Abstract

STAMP2 is a counterregulator of inflammation and insulin resistance. The aim of this study is to investigate whether activation of STAMP2 improves insulin resistance by regulating macrophage polarization in adipose tissues. The diabetic ApoE^−/−^/LDLR^−/−^ mouse model was induced by high-fat diet and low-dose streptozotocin. Samples were obtained from epididymal, subcutaneous and brown adipose tissues. Infiltration of M1/M2 macrophages and inflammatory cytokines were investigated by immunohistochemistry. We then used gene overexpression to investigate the effect of STAMP2 on macrophages infiltration and polarization and inflammatory cytokines expression. Our results showed that infiltration of macrophages, the ratio of M1/M2 macrophages and the expression of pro-inflammatory cytokines were enhanced and STAMP2 was downregulated in adipose tissues of diabetic ApoE^−/−^/LDLR^−/−^ mice compared with control mice. STAMP2 gene overexpression could significantly reduce macrophages infiltration, the ratio of M1/M2 macrophages and the expression of pro-inflammatory cytokines in epididymal and brown adipose tissues, improving insulin resistance. Our results suggested that STAMP2 gene overexpression may improve insulin resistance via regulating macrophage polarization in visceral and brown adipose tissues.

## Introduction

Insulin resistance is a major characteristic of type 2 diabetes [1]. Adipose tissue is the initial site of insulin resistance [2]. Chronic low-grade inflammation in adipose tissues plays a causal role in the pathogenesis of insulin resistance [3]. Adipose tissue consists of white and brown adipose tissues (WAT and BAT). The roles of adipose tissues in different regions in insulin resistance and the underlying mechanism that inflammation favors insulin resistance remain unclear.

Adipose tissue is closely associated with insulin resistance. Most of the previous studies focused on the roles of WAT or BAT in insulin resistance respectively. However, few studies have compared the differences of the roles of adipose tissues in different regions of the same organism in insulin resistance. It was reported that visceral and subcutaneous adipose tissues were associated with insulin resistance, especially visceral adipose tissue (VAT) [4]. Carmen reported that mice with the knockout of insulin receptors in brown adipocytes developed an insulin-secretion defect, resulting in progressive glucose intolerance [5]. The unbalance of production of pro-inflammatory and anti-inflammatory cytokines in adipose tissues is associated with insulin resistance [6], [7]. Moreover, adipose tissue macrophages (ATMs) determine the expression level of inflammatory cytokines [8], [9]. ATMs consist of at least two different phenotypes (i.e., classically activated M1 macrophages and alternatively activated M2 macrophages) [10]. The transformation of M2 to M1 macrophage and the increased M1/M2 macrophages ratio contribute to the production of pro-inflammatory cytokine [10]. These results suggest that macrophages at the crossroad of inflammation and insulin resistance might participate in the initiation and the development of insulin resistance via their polarization shift. However, the underlying mechanism of macrophage polarization remains unknown.

Recently six transmembrane protein of prostate 2 (STAMP2) has been reported as a counterregulator of inflammation and insulin resistance. Wellen reported that the visceral depot had a much stronger phenotype than the subcutaneous depot in STAMP2 deficiency in STAMP2^−/−^ mice [11]. And there is no report about STAMP2 expression in brown adipose. STAMP2 deficiency markedly increased macrophages infiltration in adipose tissues, but whether it is responsible for the macrophage polarization shift remains a question.

With the aim of evaluating the influence of STAMP2 on inflammation and macrophages infiltration of adipose tissues in different regions of diabetic animals, we constructed type 2 diabetic ApoE^−/−^/LDLR^−/−^ mouse model with STAMP2 gene overexpression in vivo, and studied the effects of STAMP2 on macrophages infiltration and polarization, inflammatory adipocytokines expression and corresponding signal pathway. We hypothesized that STAMP2 might play a major role in the mechanism of macrophage polarization shift, by which activation of STAMP2 improved insulin resistance.

## Materials and Methods

### Diabetic Model and In Vivo Experiments

Three-week-old male ApoE^−/−^/LDLR^−/−^ mice were fed a high-fat diet (34.5% fat, 17.5% protein, 48% carbohydrate; Beijing HFK Bio-Technology, China). After 6 weeks, IPGTT was performed to confirm the appearance of insulin resistance. Those mice showing insulin resistance were injected once with low dose of STZ (75 mg/kg) intraperitoneally. Two weeks after the STZ injection, most high-fat diet/STZ-treated mice displayed hyperglycemia, insulin resistance, and glucose intolerance, as previously reported [12]. At age 11 weeks, mice with similar degrees of hyperglycemia and body weight were randomly divided into vehicle (DM+vehicle, n = 6) and STAMP2-overexpression (DM+STAMP2, n = 10) groups. The mice fed a normal diet were used as nondiabetic controls, divided into Control+vehicle (n = 6) and STAMP2 overexpression (Control+STAMP2, n = 10) groups. All animal procedures were performed in accordance with animal protocols approved by Shandong University Institutional Animal Care and Use Committee.

### Intraperitoneal Glucose Tolerance Test (IPGTT)

Glucose tolerance was assessed by IPGTT after mice fasted for 12–16 h. A bolus of glucose (2 g/kg) was injected intraperitoneally, and blood samples were collected from the tail vein at 0, 15, 30, 60 and 120 min and glucose was measured using a One-Touch Glucometer (LifeScan, Milpitas, CA). The mean area under the receiver operating characteristic curve (AUC) was calculated for glucose.

### Production and Administration of Adenoviral Vector

The cDNA of mouse STAMP2 (GenBank accession no. BC006651) from SinoGenoMax Company Limited was cloned into the pShuttle vector. STAMP2 cDNA was subcloned between KpnI and EcoRI of the pShuttle expression cassette. Then recombinant pAdxsi adenovirus was constructed using the pAdxsi Adenoviral System (SinoGenoMax, Beijing, P.R. China). After amplification, viruses were purified, tittered, and stored at −80°C until used. All mice were injected via the jugular vein with 5×10^9^ plaque-forming units of virus at 20 weeks. Adenovirus transfer was repeated at 22 weeks. The control group was injected with control virus (vehicle). Four weeks after first adenovirus injection, all mice were killed for further study.

### Histological and Morphometric Analyses

Samples were taken from epididymal, subcutaneous and brown adipose tissues. Each adipose tissue sample was cut into 2 pieces. One-half of the samples were fixed in Paraformaldehyde (4%) and embedded in paraffin, and cut into 5 µm sections. A single adipocyte was measured with images captured from hematoxylin and eosin-stained sections. Every adipocyte area was assessed under ×400 magnification within adipose tissue, and a mean was obtained by quantitative morphometry with automated image analysis (Image-Pro Plus, Version 5.0; Media Cybernatics, Houston, TX).

Hepatic frozen sections (5 µm) were stained with Oil Red O (Sigma) for 10 min, washed, and then counterstained with hematoxylin for 30 s. A Nikon microscope (Nikon, Melville, NY) was used to capture the Oil Red O–stained tissue sections.

### Immunohistochemical Staining

Paraffin sections underwent immunohistochemistry by a microwave-based antigen retrieval method. The sections were incubated with primary rabbit polyclonal anti–STAMP2 (Proteintech Group Inc., Chicago, IL), anti–CD11c (BlueGene, Shanghai, China), anti-CD206 (BlueGene, Shanghai, China), anti-IL-6, anti-TNF-α**,** anti-MCP-1, anti-IL-10 and primary rat polyclonal anti-F4/80 antibodies (Abcam, Cambridge, MA, USA) overnight and then with a matching biotinylated secondary antibody for 30 min at 37°C. Negative controls were omission of the primary antibody. The stained sections were developed with diaminobenzidine and counterstained with hematoxylin. The results were viewed under a confocal FV 1000 SPD laser scanning microscope (Olympus, Japan).

### Western Blot

Western blot analysis was as previously described [13]. We used antibodies against STAMP2 (Proteintech Group Inc., Chicago, IL), p-Jun NH2-terminal kinase (JNK)/JNK (Cell Signaling Technology, Beverly, MA), followed by anti-IgG horseradish peroxidase-conjugated secondary antibody. STAMP2 protein level was normalized to that of β-actin as an internal control and phosphorylated proteins to that of total protein.

### Statistical Analysis

Values are presented as mean ± SEM. SPSS 17.0 (SPSS, Chicago, IL) was used for statistical analysis. Results were compared by one-way ANOVA, followed by Tukey-Kramer post hoc test and independent samples *t* test. A *P*<0.05 was considered statistically significant.

## Results

### Generation of Type 2 Diabetic Mouse Model

**Figure 1:**
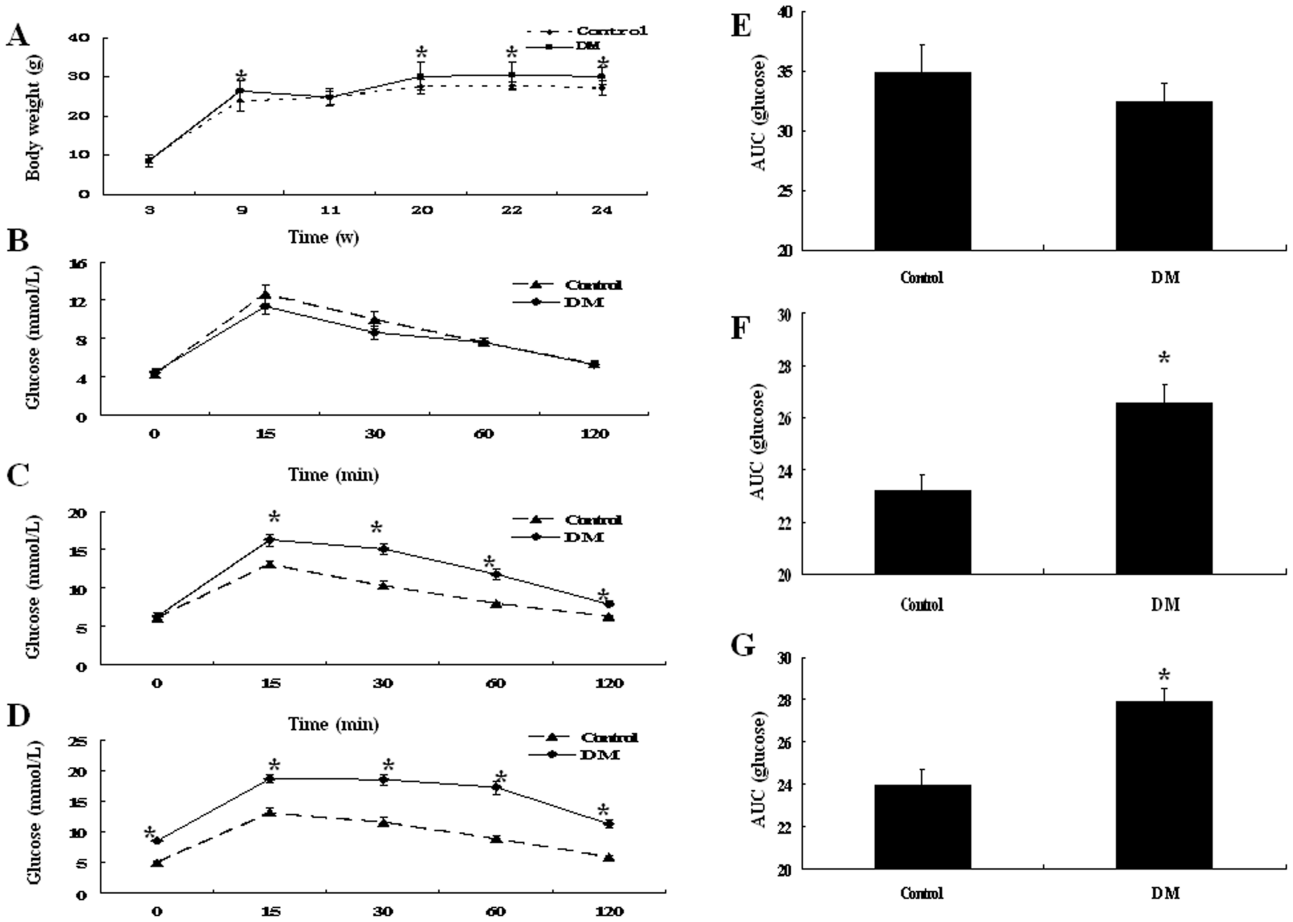
Body weight and IPGTT in ApoE^−/−^/LDLR^−/−^ mice. A: Body weight at the age of 3, 9, 11, 20, 22 and 24 weeks. B–D: Glucose tolerance tests at the age of 3, 9 and 11 weeks. E–G: The area under blood glucose concentration curve (AUC) at the age of 3, 9 and 11 weeks. Data are mean ± SEM; n = 16 per group. *P<0.05 vs. Control.

At baseline, there were no significant differences in the levels of blood glucose between the control and diabetic mice confirmed by IPGTT (Fig. 1*B* and *E*). Insulin resistance was induced after a 6-week high-fat diet. By IPGTT, the levels of blood glucose in the diabetic group were significantly higher at week 9 than the control group at all of the time points texted except at 0 min (*P*<0.05) (Fig. 1*C*). The AUC across the time for glucose level was higher at week 9 than baseline (*P*<0.05) (Fig. 1*F*). And two weeks after the STZ injection, compared with the control group, the diabetic group showed impaired glucose tolerance on IPGTT; blood glucose levels especially were significantly elevated at all time points compared with the control (*P*<0.05) (Fig. 1*D* and *G*). The mean body weight was significantly higher for the diabetic group than the control group at the age of 9, 20, 22 and 24 weeks (*P*<0.05) (Fig. 1*A*). Thus, the diabetic group showed typical type 2 diabetic features of insulin resistance and obesity that could insist throughout the experiment after high-fat diet and STZ injection.

### Overexpression of STAMP2 in WAT and BAT

**Figure 2:**
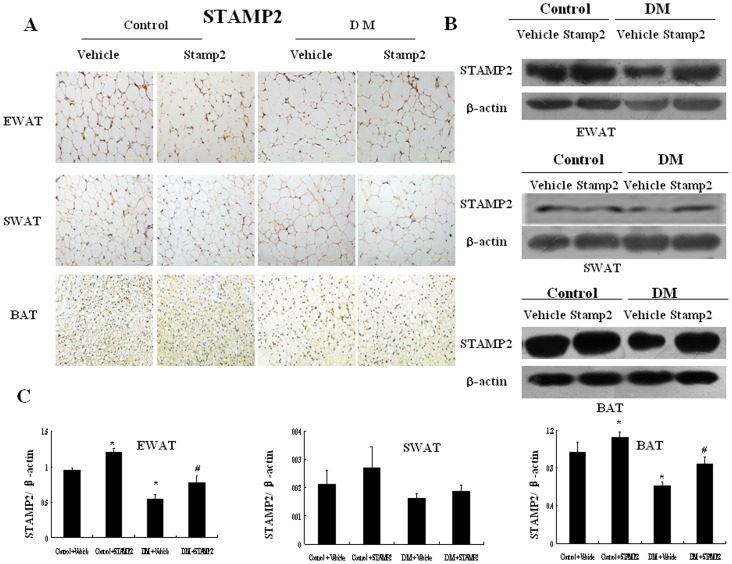
Images and quantifications of STAMP2 expression in adipose tissues of ApoE^−/−^/LDLR^−/−^ mice with or without STAMP2 overexpression. A: Immunohistochemical staining for adipose tissue STAMP2 (brown staining considered positive staining; scale bar: 50 µm); B. Representative Western blot of adipose tissue STAMP2; C. Western blot analysis of STAMP2 expression. Data are mean ± SEM; *P<0.05 vs. Control+vehicle; ^#^ *P*<0.05 vs. DM+vehicle. EWAT: epididymal white adipose tissue; SWAT: subcutaneous white adipose tissue; BAT: brown adipose tissue.

Compared with control group, endogenous STAMP2 expression was significantly decreased in epididymal white adipose tissue (EWAT) and BAT in diabetic ApoE^−/−^/LDLR^−/−^ mice (*P*<0.001) (Fig. 2*A–C*). And overexpression of STAMP2 could significantly increase STAMP2 expression in EWAT and BAT in diabetic ApoE^−/−^/LDLR^−/−^ mice (*P*<0.001) (Fig. 2 *A–C*). However, there were no significant differences in STAMP2 expression in subcutaneous white adipose tissues (SWAT) in all groups.

### WAT and BAT Morphology

**Figure 3:**
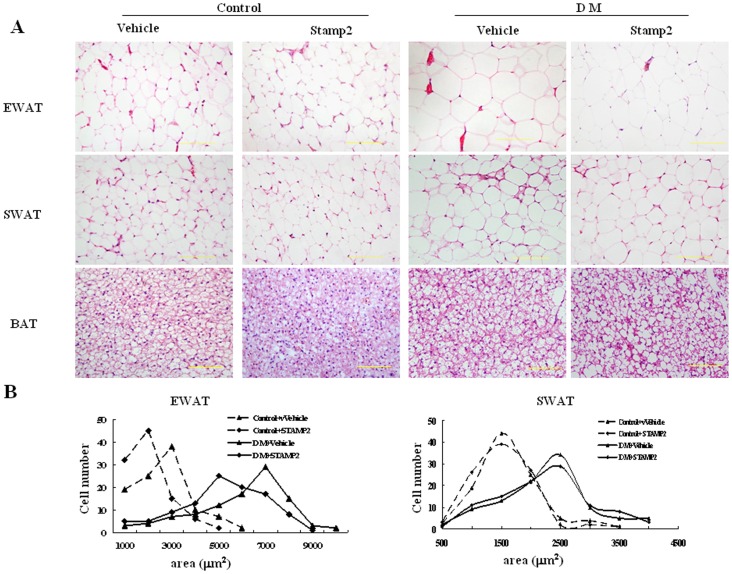
The effect of STAMP2 gene overexpression on adipose tissue morphology in ApoE^−/−^/LDLR^−/−^ mice. A: Adipose tissue sections stained with hematoxylin and eosin; B: Adipocyte cell size measured under light microscopy (three sections per mice; n = 4 per group; scale bar: 50 µm). EWAT: epididymal white adipose tissue; SWAT: subcutaneous white adipose tissue; BAT: brown adipose tissue.

Hematoxylin and eosin (H&E) staining of EWAT and SWAT sections revealed significant increases in adipocyte cell size in diabetic ApoE^−/−^/LDLR^−/−^ mice (Fig. 3*A*–*B*). Compared with the diabetic group, the epididymal adipocyte cell size was significantly decreased in STAMP2 overexpression group (Fig. 3 *A*–*B*). There was no significant difference in adipocyte size in SWAT between the diabetic and STAMP2 overexpression groups.

H&E stained BAT from the diabetic ApoE^−/−^/LDLR^−/−^ mice showed larger lipid vacuoles compared to the smaller vacuoles in control group. White adipocytes in BAT were seen in the diabetic group. Compared with the diabetic group, STAMP2 overexpression group showed smaller vacuoles and reduced white adipocytes in BAT (Fig. 3*A*).

### STAMP2 Gene Overexpression Reduced Macrophages Infiltration and the Ratio of M1/M2 Macrophages

**Figure 4:**
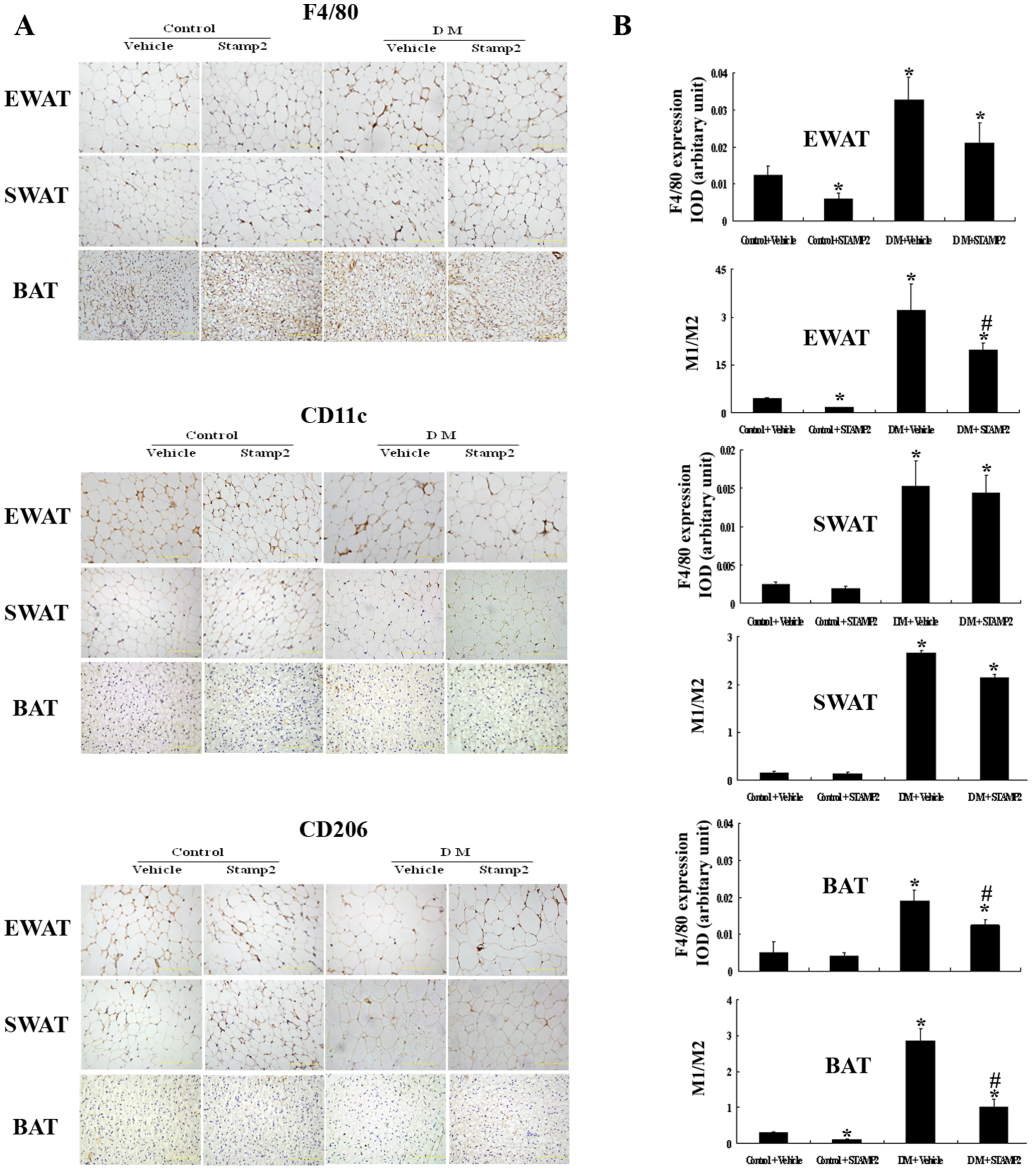
The effect of STAMP2 gene overexpression on macrophages infiltration and macrophage polarization in WAT and BAT of ApoE^−/−^/LDLR^−/−^ mice. A: Immunohistochemical staining showing accumulation of F4/80-positive, CD11c-positive and CD206-positive cells in adipose tissue (brown staining considered positive staining; scale bar: 50 µm); B: Semiquantification of F4/80 immunnohistochemical staining and analysis of the ratio of CD11c-positive cells to CD206-positive cells. Data are mean ± SEM (three sections per mice; n = 4 per group). *P<0.05 vs. Control+vehicle; ^#^ *P*<0.05 vs. DM+vehicle. IOD: integrated optical density; EWAT: epididymal white adipose tissue; SWAT: subcutaneous white adipose tissue; BAT: brown adipose tissue.

The number of infiltrating macrophages in WAT was significantly increased in the diabetic group than Control+vehicle, Control+STAMP2 and DM+STAMP2 groups. The numbers of M1 and M2 macrophages using CD11c and CD206 as M1 and M2 markers respectively were significantly increased in WAT in the diabetic group. The ratio of M1/M2 macrophages was elevated in WAT in the diabetic group (*P*<0.05) (Fig. 4*B*). STAMP2 overexpression group showed significantly decreased M1/M2 macrophages ratio in EWAT. We also found that infiltration of macrophages and the ratio of M1/M2 macrophages were elevated significantly in BAT in the diabetic ApoE^−/−^/LDLR^−/−^ group (*P*<0.05) (Fig. 4*B*). STAMP2 overexpression group showed significantly decreased macrophages infiltration and M1/M2 macrophages ratio in BAT. Those changes above were not seen in SWAT in STAMP2 overexpression group. These data suggested that STAMP2 overexpression induced the transformation of M1 to M2 macrophage in EWAT and BAT.

**Figure 5:**
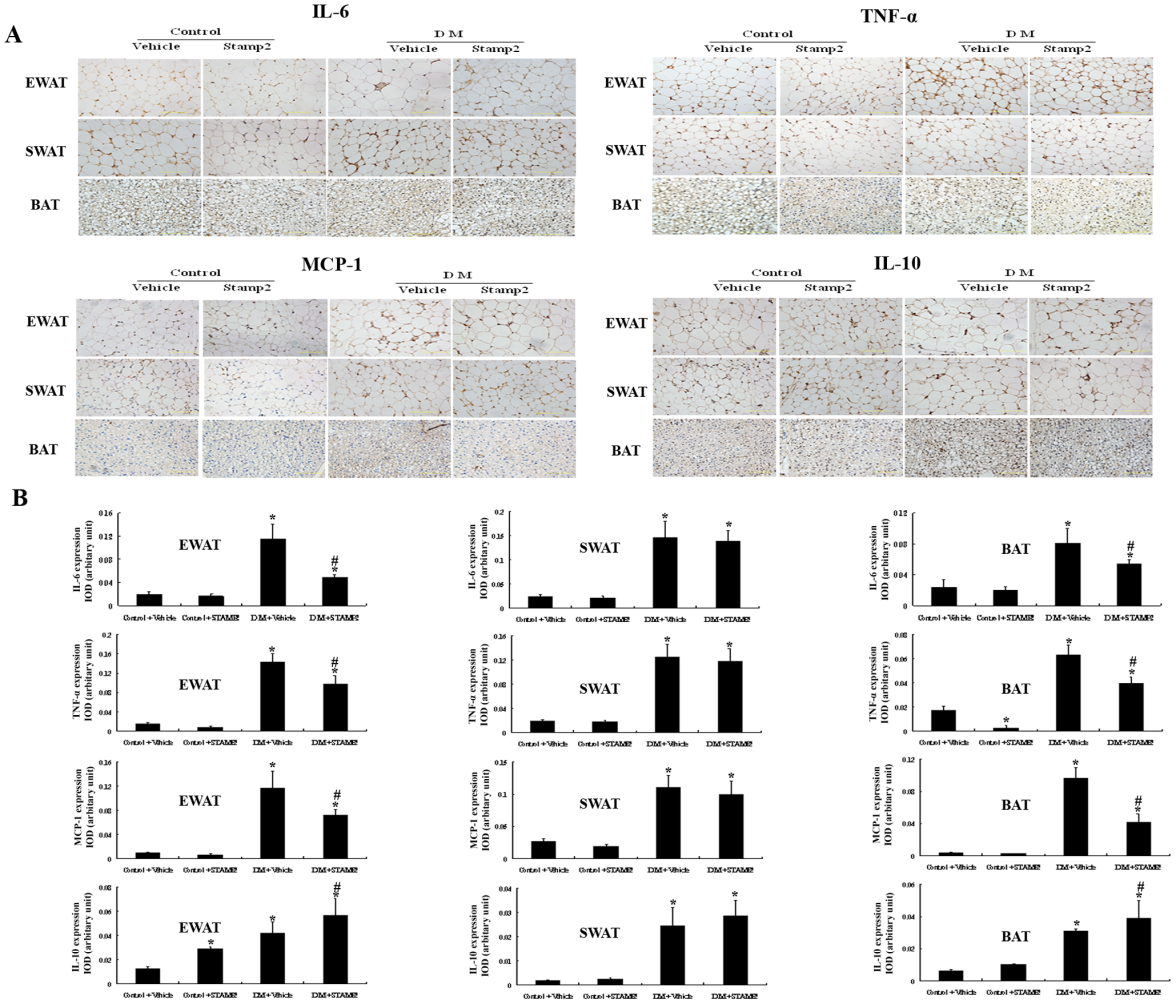
The effect of STAMP2 gene overexpression on inflammatory factors expression in WAT and BAT of ApoE^−/−^/LDLR^−/−^ mice. A: Immunohistochemical staining for adipose tissue IL-6, TNF-α, MCP-1 and IL-10 (brown staining considered positive staining; scale bar: 50 µm). B: Semiquantification of IL-6, TNF-α, MCP-1 and IL-10 immunohistochemical staining. Data are mean ± SEM (three sections per mice; n = 4 per group). *P<0.05 vs. Control+vehicle; ^#^ *P*<0.05 vs. DM+vehicle. IOD: integrated optical density; EWAT: epididymal white adipose tissue; SWAT: subcutaneous white adipose tissue; BAT: brown adipose tissue.

We also investigated the expression of inflammatory cytokines. The MCP-1, IL-6 and TNF-α, pro-inflammatory cytokines secreted by M1 macrophages, were upregulated in WAT and BAT in diabetic ApoE^−/−^/LDLR^−/−^ mice compared with those in control. The IL-10, anti-inflammatory cytokine produced by M2 macrophages, was also upregulated in WAT and BAT in diabetic ApoE^−/−^/LDLR^−/−^ mice. STAMP2 overexpression group showed decreased pro-inflammatory cytokines and increased anti-inflammatory cytokine levels in EWAT and BAT compared with the diabetic group (*P*<0.05) (Fig. 5*B*). There were no significant differences in the expression of inflammatory factors in SWAT between the diabetic and STAMP2 overexpression groups. Our results suggested that STAMP2 overexpression decreased pro-inflammatory factors and the ratio of M1/M2 macrophages in EWAT and BAT in ApoE^−/−^/LDLR^−/−^ mice.

### STAMP2 Gene Overexpression Improved Glucose Tolerance

**Figure 6:**
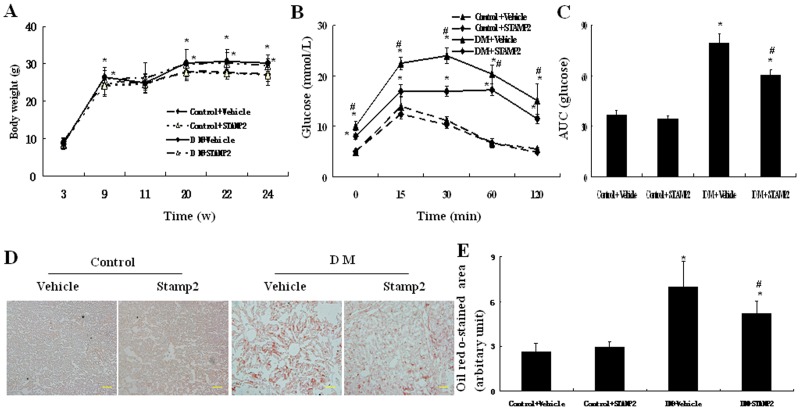
The effect of STAMP2 gene overexpression on body weight, IPGTT and aberrant lipid accumulation in liver of ApoE^−/−^/LDLR^−/−^ mice. A: Body weight at the age of 3, 9, 11, 20, 22 and 24 weeks. B: Glucose tolerance tests at the age of 24 weeks. C: The area under blood glucose concentration curve (AUC) at the age of 24 weeks. D: Representative Oil Red O–stained adipose tissue sections (scale bar: 20 µm). E: Semiquantification of Oil Red O staining. Data are mean ± SEM of six independent observations in each group. *P<0.05 vs. Control+vehicle; ^#^ *P*<0.05 vs. DM+vehicle.

At the end of the experiment, although the diabetic and DM+STAMP2 groups showed higher body weight than the control group, STAMP2 overexpression could not decrease body weight of the diabetic mice (Fig. 6*A*). Compared with the diabetic group, DM+STAMP2 group showed significantly lower blood glucose levels and mean AUC on IPGTT (*P*<0.05) (Fig. 6*B* and *C*). These data suggested that STAMP2 overexpression improved insulin resistance in diabetic ApoE^−/−^/LDLR^−/−^ mice.

### STAMP2 Gene Overexpression Reduced Aberrant Lipid Accumulation

We evaluated the lipid accumulation in liver in each group. Aberrant lipid accumulation could be seen in diabetic ApoE^−/−/^LDLR^−/−^ mice. STAMP2 overexpression could markedly reduce aberrant lipid accumulation within liver in diabetic ApoE^−/−/^LDLR^−/−^ mice (*P*<0.05) (Fig. 6*D* and *E*).

### STAMP2 Gene Overexpression Inhibited JNK Pathway

**Figure 7:**
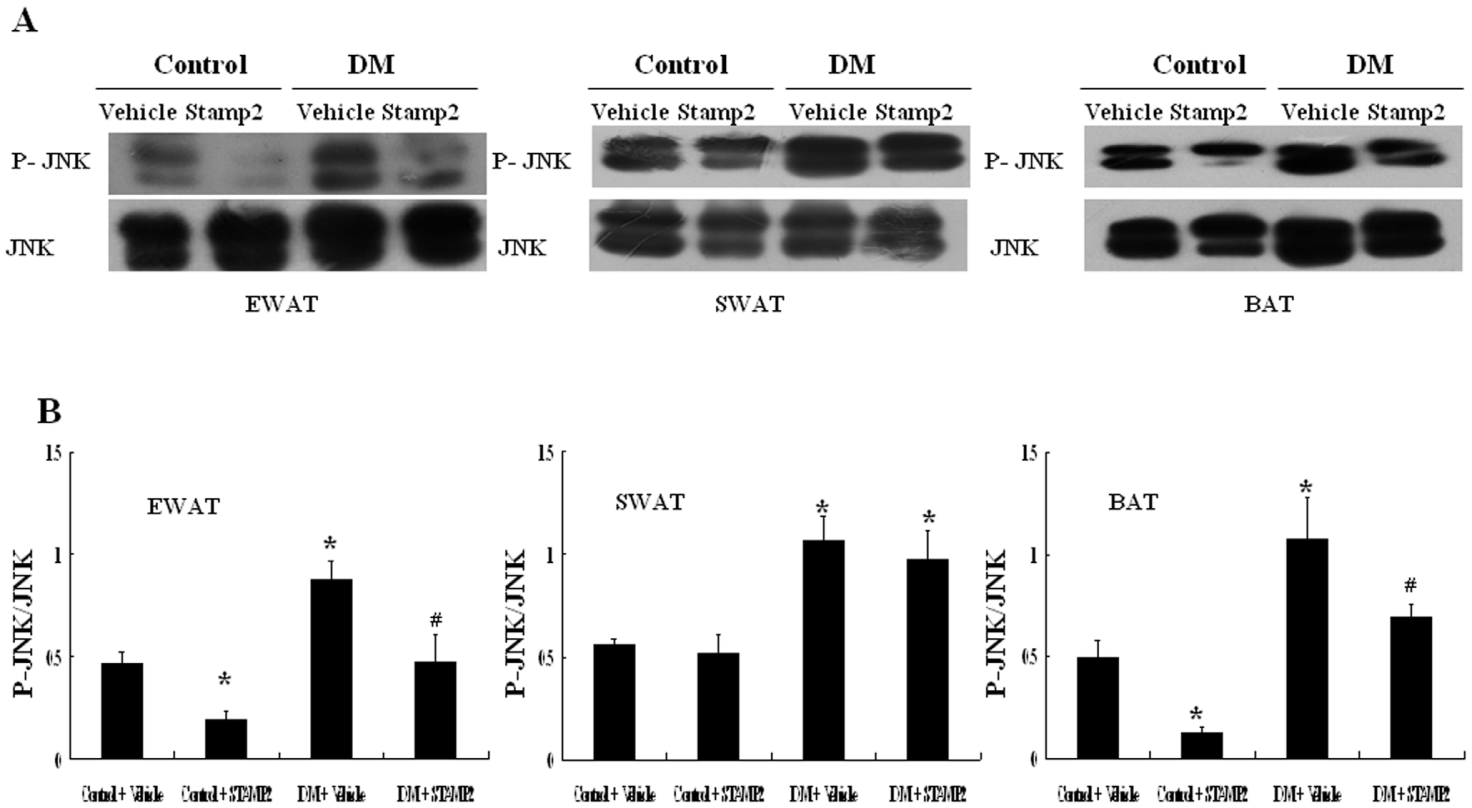
The effect of STAMP2 gene overexpression on the phosphorylation of JNK in WAT and BAT of ApoE^−/−^/LDLR^−/−^ mice. A: Representative Western blot of adipose tissue P-JNK and JNK; B: Western blot analysis of P-JNK/JNK. Data are mean ± SEM. *P<0.05 vs. Control+vehicle; ^#^ *P*<0.05 vs. DM+vehicle. EWAT: epididymal white adipose tissue; SWAT: subcutaneous white adipose tissue; BAT: brown adipose tissue.

With STAMP2 overexpression treatment, the phosphorylation of JNK was abolished by 55% both in EWAT and BAT in the diabetic group (Fig. 7*B*). Thus, STAMP2 appeared to have the effect on inhibiting JNK activation.

## Discussion

In this study of diabetic ApoE^−/−^/LDLR^−/−^ mice, we found that infiltration of macrophages and the ratio of M1/M2 macrophages were significantly increased in adipose tissues in different regions. STAMP2 was markedly downregulated in VAT and BAT, with no significant change in SWAT. STAMP2 overexpression could decrease macrophages infiltration and promote its transformation from M1 to M2 macrophage in adipose tissues, and thus improve insulin resistance.

Adipose tissue consists of white and brown adipose tissues (WAT and BAT). WAT is specialized to store energy with the form of triglyceride, and BAT is involved in the dissipation of energy via heat generation. It has been suggested that an increased adipocyte size in WAT is associated with insulin resistance [14]. Increased fat cell size may represent the failure of the adipose tissue mass to expand and therefore to accommodate an increased energy influx [15]. Our study found that the sizes of adipocytes in VAT and SWAT were significantly increased in diabetic ApoE^−/−^/LDLR^−/−^ mice. Larger fat cells secrete more inflammatory factors, and a significant increase in TNF-α expression was seen in WAT in diabetic ApoE^−/−^/LDLR^−/−^ mice. TNF-α inhibited adipogenesis [16], and the inability for adipogenesis may promote adipocyte hypertrophy, contributing to insulin resistance [17], [18]. In this study, increased TNF-α expression was also seen in BAT in diabetic ApoE^−/−^/LDLR^−/−^ mice. TNF-α induces insulin resistance in BAT [19] and directly inhibits the expression of uncoupling protein-1(UCP1) which is vital for the regulation of body temperature [20], [21]. The combination of adipocyte hypertrophy in WAT and decreased energy expenditure in BAT causes and aggravates insulin resistance. Therefore, decreasing inflammation level, shrinking white fat cell size and increasing BAT energy expenditure may be crucial for improving insulin resistance.

The expression levels of inflammatory cytokines are determined by macrophages infiltration [10]. Macrophages infiltration within WAT was possible to affect fat expansion through a paracrine action on adipocyte differentiation, and thus may indirectly contribute to insulin resistance [22]. In this study, macrophages infiltration was significantly increased and adipocyte hypertrophy was obvious in WAT of diabetic ApoE^−/−^/LDLR^−/−^ mice. Wellen et al. reported that activation of STAMP2 could reduce infiltration of macrophages in VAT in STAMP2^−/−^ mice [11], which was further confirmed by our study through STAMP2 gene overexpression. However, the expression of pro-inflammatory cytokines is determined by the ratio of M1/M2 macrophages, and the ratio of M1/M2 macrophages is better correlated with insulin resistance [10]. There is no further research on the effect of STAMP2 on macrophage polarization. Our study demonstrated that STAMP2 overexpression could significantly decrease the ratio of M1/M2 macrophages in VAT.

In this study, we observed the phenomenon of infiltration of white adipocytes in BAT in diabetic ApoE^−/−^/LDLR^−/−^ mice, which was a kind of adaption for energy storage. The expression of STAMP2 was significantly decreased in BAT, too. With STAMP2 overexpression, the ratio of M1/M2 macrophages, the expression of pro-inflammatory cytokines and infiltration of white adipocytes in BAT were significantly decreased. Therefore, we supposed that activation of STAMP2 may contribute to insulin resistance via decreasing infiltration of macrophages, especially the ratio of M1/M2 macrophages in VAT and BAT. As indicated above, STAMP2 was the key point to be at the crossroad of inflammation and insulin resistance and may play a major role in the mechanism of macrophage polarization shift.

Adipose tissues in different regions have different roles in the process of insulin resistance. The functional and metabolic properties of adipose tissue from different sites differ significantly. VAT contains more inflammatory cell types (e.g. macrophages) and shows an increased inflammatory response to factors released from macrophages than SWAT, leading to obesity related diabetes [23]. BAT is involved in the dissipation of energy via heat generation. TNF-α induces insulin resistance in BAT [19] and directly inhibits BAT energy expenditure [20], [21], aggravating insulin resistance. Therefore, inflammation plays a key role in insulin resistance. STAMP2 could regulate inflammation and the expression levels of STAMP2 were lower in SWAT than VAT and BAT in control and diabetic ApoE^−/−^/LDLR^−/−^ mice. Correspondingly, STAMP2 overexpression could decrease inflammatory factors in VAT and BAT but not in SWAT, which was consistent with Wellen’s report. Together, these data proved that lower expression of STAMP2 in SWAT might lead to no significant correlation between SWAT and insulin resistance as compared with VAT [24]. STAMP2 at the crossroad of inflammation and insulin resistance might be involved in the regional differences of adipose tissues in insulin resistance.

JNK is a crucial mediator of insulin resistance and JNK1 activation in adipose tissue can cause insulin resistance [25]. In mice with adipocyte-selective deletion of JNK1, increased insulin activity was seen [25]. In our study, the ratio of P-JNK1/JNK1 was increased in WAT and BAT in diabetic ApoE^−/−^/LDLR^−/−^ mice. With STAMP2 overexpression, the ratios of P-JNK1/JNK1 in EWAT and BAT, but not in SWAT were significantly decreased. Therefore, we supposed that activation of STAMP2 may regulate macrophage polarization via inhibiting JNK1 signaling pathway to reduce the expression of pro-inflammatory cytokines, and thus improve insulin resistance.

In addition, STAMP2 gene overexpression significantly improved glucose tolerance in diabetic ApoE^−/−^/LDLR^−/−^ mice. Therefore, we supposed that STAMP2 gene overexpression could improve insulin resistance, which was obvious in VAT and BAT. Besides the reduction of macrophage infiltration in adipose tissues, the benefit of STAMP2 overexpression was mainly due to the decrease of the ratio of M1/M2 macrophage, which involved the possible signaling mechanism about the inhibition of JNK pathway to reduce inflammation.

Our study contains some limitations. Firstly, the STAMP2 regulates insulin resistance in ApoE and LDLR-dual KO diabetic mice, further studies with wild type diabetic mice have to determine whether the effects of STAMP2 on insulin resistance depend on the presence of ApoE and LDLR. Moreover, the expression levels of STAMP2 in SWAT are much lower than that in EWAT and BAT of the controls and DM mice. It appears that some specific inhibitors or cellular machinery in SWAT tissues could downregulate the expression or increase the clearance of STAMP2 in SWAT tissues. More experiments should be considered to elucidate the mechanism in the future.

In conclusion, STAMP2 gene overexpression may improve insulin resistance via regulating macrophage polarization in visceral and brown adipose tissues, implicating its potential role in the mechanism of macrophage polarization shift in adipose tissues and the treatment of insulin resistance.
