# Correction: Overexpressing STAMP2 Improves Insulin Resistance in Diabetic ApoE^−/−^/LDLR^−/−^ Mice via Macrophage Polarization Shift in Adipose Tissues

**DOI:** 10.1371/annotation/39606c88-c2a1-45e7-8b2d-9b8f7df6f05f

**Published:** 2013-12-31

**Authors:** Lu Han, Meng-Xiong Tang, Yun Ti, Zhi-Hao Wang, Jia Wang, Wen-Yuan Ding, Hua Wang, Yun Zhang, Wei Zhang, Ming Zhong

Affiliations for multiple authors are incorrect. Please view the list of authors with the correctly indicated list of affiliations below:

Lu Han^1^, Meng-Xiong Tang^1,2^, Yun Ti^1^, Zhi-Hao Wang^1,3^, Jia Wang^1^, Wen-Yuan Ding^1^, Hua Wang^1^, Yun Zhang^1^, Wei Zhang^1^, Ming Zhong^1^* 

1 Key Laboratory of Cardiovascular Remodeling and Function Research Chinese Ministry of Education and Chinese Ministry of Public Health, Department of Cardiology, Qilu Hospital of Shandong University, Ji’nan, People’s Republic of China

2 Department of Emergency, Qilu Hospital of Shandong University, Ji’nan, People’s Republic of China

3 Department of Geriatric Medicine, Qilu Hospital of Shandong University, Ji’nan, People’s Republic of China

